# Cost-utility analysis of 1-year treatment with adalimumab/standard care and standard care alone for ulcerative colitis in Poland

**DOI:** 10.1007/s00228-016-2103-4

**Published:** 2016-08-06

**Authors:** Ewa Stawowczyk, Paweł Kawalec, Andrzej Pilc

**Affiliations:** 1 StatSoft Polska Sp. z o.o., Krakow, Poland; 2 Department of Drug Management, Institute of Public Health, Faculty of Health Sciences, Jagiellonian University Medical College, 20, Grzegórzecka street, 31-531 Kraków, Poland; 3 Department of Neurobiology, Institute of Pharmacology, Polish Academy of Sciences, Krakow, Poland

**Keywords:** Ulcerative colitis, Cost-utility, Adalimumab, Economic analysis, Indirect costs

## Abstract

**Purpose:**

Until recently, surgery was the only remaining choice for moderate to severe chronic ulcerative colitis patients who failed standard treatment or when it was not tolerated. Anti-TNFα treatment is a new, non-invasive option for the management of ulcerative colitis. The objective of this study was to assess the cost-effectiveness of induction and maintenance treatment up to 1 year of ulcerative colitis with adalimumab/standard care and standard care alone in Poland.

**Methods:**

A Markov model was used to estimate the expected costs and effects of adalimumab/standard care and a standard care alone. For each treatment option, the costs and quality adjusted life years were calculated to estimate the incremental cost-utility ratio. The analysis was performed from the perspective of the Polish public payer and society over a 30-year time horizon. Different direct and indirect costs and utility values were assigned to the various model health states.

**Results:**

The treatment of ulcerative colitis patients with adalimumab/standard care up to 1 year instead of a standard care alone resulted in 0.14 additional years of life with full health (QALYs). The incremental cost-utility ratio of adalimumab/standard care compared to the standard care alone is estimated to be 76,120 €/QALY gained from NHF perspective and 71,457 €/QALY gained from social perspective.

**Conclusions:**

The biologic treatment of ulcerative colitis patients with adalimumab/standard care is more effective but also more costly compared with standard care alone.

**Electronic supplementary material:**

The online version of this article (doi:10.1007/s00228-016-2103-4) contains supplementary material, which is available to authorized users.

## Introduction

Ulcerative colitis (UC) is an idiopathic inflammatory bowel disorder characterized by an inflammatory reaction involving the colonic mucosa [1, 2]. The clinical course is unpredictable and marked by alternating periods of exacerbation and remission, which may occur spontaneously or in response to treatment changes or intercurrent illnesses [3, 4]. Although progress has been made in the overall management of the disease, no medical cure has been discovered [5].

The introduction of anti-tumor necrosis factor-alpha (anti-TNFα) treatment allowed a new option for the management of ulcerative colitis and is expected to decrease the rate of colectomies or at least to extend the time to surgery, compared with standard treatment. Adalimumab/standard care superior efficacy compared to standard care alone in moderate to severe non-acute UC has been well established by the clinical trials [6, 7]. On the other hand, the use of biologics constitutes a heavy burden for the public payer, so its usage can be limited in many countries.

In Poland, patients with severe UC who are not able to have cyclosporine therapy and do not respond to standard care have the possibility to receive the induction treatment with infliximab, which consists of three administrations of the drug. At present, there is no biological maintenance treatment of ulcerative colitis reimbursed in Poland, hence patients often lose their response or remission, which were achieved during the induction phase. Additionally, the lack of biological maintenance treatment leads to an increased rate of colectomies. Adalimumab, nor any other biologic therapy, is not reimbursed from public funds in UC treatment at all. In this connection, there was a need for economic evaluation of UC induction and maintenance therapy with a TNFα inhibitor at Polish settings.

This study uses an economic evaluation to assess the cost-effectiveness of induction and maintenance treatment up to 1 year of ulcerative colitis with adalimumab/standard care and standard care alone in Poland.

## Methods

### Overview

**Figure 1:**
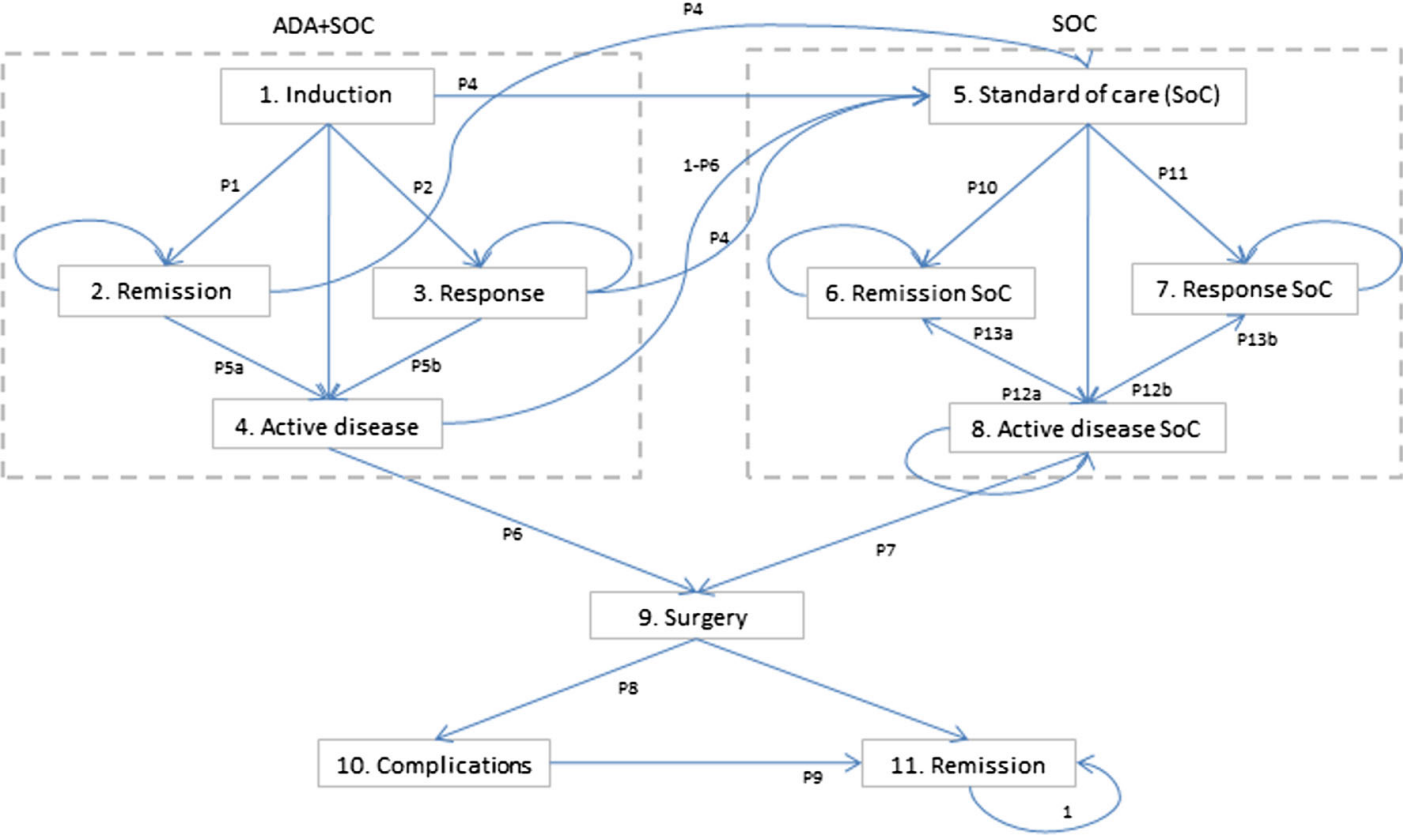
Structure of Markov model for patients with UC. The cycle length during induction phase is 1 week, from week 9, it is 8 weeks. All complications after surgery (state 10.) are assumed to be temporary and resolved during the 8-week period. It was assumed that the probability of death will be the same for each clinical state. *ADA* adalimumab, *SoC* standard of care

A Markov model was used to estimate the expected costs and effects of adalimumab/standard care and standard care alone used in the induction and maintenance treatment of moderate to severe ulcerative colitis (model structure, inputs, transition probabilities, costs of health states, and utilities are presented in Fig. 1, Tables 1 and 2). For each treatment option, the costs and quality adjusted life years (QALYs) were calculated to estimate the incremental cost-utility ratio (ICUR). The analysis was taken from the perspective of the Polish public payer and also from expanded social perspective (indirect costs included). Ulcerative colitis could be a lifelong disease, which is why the 30-year time horizon was selected for the base-case analysis. Costs and outcomes were discounted at an annual rate of 5 and 3.5 %, respectively.

**Table 1:** Clinical inputs and utilities

Parameter		Value	95 % LCI/ minimum value	95 % UCI/ maximum value	Reference
Response rate—8 week	Adalimumab/standard care (RR)	1.34	1.02	1.77	[6]
	Standard care alone	0.25	0.20	0.31	[6]
Remission rate—8 week	Adalimumab/standard care (RR)	1.77	1.10	2.86	[6]
	Standard care alone	0.09	0.06	0.13	[6]
The probability of response per cycle—52 week	Standard care alone	0.016	0.010	0.023	[6]
Response per cycle—52 week (RR)	Adalimumab/standard care	1.32	0.80	2.18	[6]
The probability of remission per cycle—52 week	Standard care alone	0.014	0.009	0.020	[6]
Remission per cycle—52 week (RR)	Adalimumab/standard care	2.03	1.24	3.32	[6]
The probability of response loss per cycle—8–52 weeks	Adalimumab/standard care	0.161	–	–	[6]
	Standard care alone	0.158	–	–	[6]
The probability of remission loss per cycle—8–52 weeks	Adalimumab/standard care	0.000	–	–	[6]
	Standard care alone	0.016	–	–	[6]
The probability of complications after surgery		0.53	0.27	0.53	[11]
Surgery rate per cycle	Adalimumab/standard care (RR)	0.77	0.33	1.86	[10]
	Standard care alone	0.75 %	–	–	[10]
Utilities	Active treatment	0.420	0.320	–	[12, 13]
	Remission	0.880	0.790	0.910	12, 13]
	Response	0.760	0.580	0.940	[12]
	Remission after surgery	0.610	–	–	[12]
	Complications after surgery	0.420	–	0.490	[12, 13]

*RR* relative risk, *LCI* lower confidence interval, *UCI* upper confidence interval

**Table 2:** Cost inputs

Parameter		Mean	95 % LCI	95 % UCI	Reference
Drug cost [PLN]	Adalimumab 1 mg	54.55	–	–	Decree of the Minister of Health
	Azathioprine 1 mg	0.0107	–	–	
	Prednisolone 1 mg	0.1055	–	–	
	Mesalazine 1 mg	0.0015	–	–	
	Mercaptopurine 1 mg	0.0166	–	–	
Monitoring costs per cycle [PLN]	8 weeks cycle	121.56	–	–	Expert opinion, decree of the President of NHF
	1 week cycle	15.20	–	–	
Administration costs [PLN]		468.00	–	–	Expert opinion, decree of the President of NHF
Surgery cost [PLN]		12,480	6240	37,440	Expert opinion, decree of the President of NHF
Complication after surgery treatment [PLN]		4160	–	–	Expert opinion, decree of the President of NHF
Standard treatment per cycle [PLN]	8 weeks cycle	204.32	–	–	Decree of the Minister of Health; expert opinion
	1 week cycle	25.54	–	–	
Indirect costs [PLN/year]	Remission	6523.75	–	–	Data unpublished
	Active disease	22,934.58	–	–	

€1 = 4.2 PLN, based on the average exchange course from the year 2015

*PLN* Polish zloty, *NHF* National Health Fund

The target population consists of a hypothetical cohort of adult patients with moderately to severely active ulcerative colitis, despite concurrent therapy with steroids and/or azathioprine or 6-mercaptopurine. The average weight and age of a patient was 75.37 kg (range: 73.60–77.14) and 39.60 years (95 % CI: 38.05–41.15), respectively, and the percentage of women was 42.7 % (95 % CI: 35.6–45.5 %). This was based upon baseline data from the reference clinical trial ulcerative colitis long-term remission and maintenance with adalimumab 2 (ULTRA 2) [6]. Based on the current practice and reference clinical trial, in the model adalimumab was assumed to be administered in a dose of 160 mg at week 0, 80 mg at week 2, and 40 mg every other week beginning at week 4 up to 1 year [6, 7].

### Model structure

The modeling was carried out based on a Markov-type cohort simulation process and implemented in Microsoft Excel 2007 with Visual Basic for Applications tool (Microsoft Corporation, Redmond, WA). The patient enters this model when starting the induction treatment. The time horizon is divided into two periods: from week 0 to 8 (period 1; induction treatment) and the weeks from nine (period 2; maintenance treatment). The cycle length during period 1 is 1 week, and cycles in period 2 last 8 weeks. After the ninth cycle, the response to induction therapy and remission was assessed and biological treatment was continued only in responders (patients who responded or experienced remission). Mayo score was used to assess the UC activity (scores can range from 0 to 12, with higher scores indicating more severe disease activity): values from 0 to 2 means remission, 3–5 mild disease, 6–12 moderate-severe disease [6]. The clinical response was defined as a decrease from the baseline in the total Mayo score by at least 3 points and at least 30 %, with an accompanying decrease in rectal bleeding subscore of at least 1 point or an absolute rectal bleeding subscore of 0 or 1. Clinical remission was defined as a total Mayo score of 2 points or lower, with no individual subscore exceeding 1 point [6]. In accordance with Polish clinical practice, for standard care, it is assumed that in both the induction and maintenance phases, 100 % of patients have both corticosteroids and aminosalicylates, 80 % have mercaptopurine, and 20 % have azathioprine.

Patients who have neither remission nor response to induction treatment with the TNFα inhibitor/standard care or standard care alone will stop the treatment, move to an active disease state and start standard care alone or will have a colectomy. If one of the treatments (adalimumab/standard care or standard care alone) led to a clinical response or remission, the patient continued with the treatment in the maintenance phase.

Maintenance treatment with adalimumab is restricted to 1 year in base-case analysis and no limitation for biological treatment was assumed in sensitivity analysis (adalimumab administered until loss of response or death, according to what occurs first). Patients who experienced the response or remission can sustain or lose it during the next cycle. The treatment can be discontinued when unacceptable adverse events occur; in this case, the patient moves to a standard care alone state. Patients who failed adalimumab/standard care or standard care alone treatment continued standard care in the maintenance phase, but they could have a colectomy if their disease remained active. Patients can experience remission or response during standard care, the same as during standard care alone treatment. Standard care is continued regardless of whether the patient has remission, response, or is in an active disease state. All patients who had a colectomy can experience temporary complications and finally achieve clinical remission after surgery (see: Fig. 1.). It was assumed that all complications occur immediately after surgery (in the same cycle) and are resolved during the 8-week period.

Certain adverse events were not included in the model. In accordance with reference clinical trial [6], adalimumab treatment was generally well tolerated and the overall safety profile of adalimumab was comparable with that of placebo. A similar proportion of patients in each study group experienced treatment emergent adverse events, which were nonserious, mild, or moderate in severity, and were considered not related or probably not related to study drug [6].

There is no evidence that patients with UC have lower life expectancy; thus, the probability of death was calculated on a basis of life expectancy table for general Polish population (www.stat.gov.pl). It was assumed that the probability of death will be the same for each clinical state. No data was found on the different probability of death from particular clinical states of model for natural course of the disease.

### Clinical inputs

Transition probabilities in the model were calculated based on the response, remission rates, and discontinuation due to adverse event rates which came from randomized, double-blind, placebo-controlled trial. The ULTRA 2 study evaluated the efficacy of adalimumab in induction and maintenance of clinical remission in patients with moderate to severe ulcerative colitis who received concurrent treatment with oral corticosteroids or immunosuppressants [6]. As the definitions of remission and response were overlapping in ULTRA 2 study [6], responders from this analysis excluded those who achieved remission. The above provides the separation of these two states in a model.

In ULTRA 2 study, the effectiveness was assessed at week 8 (after induction treatment), at week 32, and at week 52 (maintenance treatment; data available only for week 8 and week 52 [6]). Transition probabilities for adalimumab/standard care after discontinuation of the biological treatment were assumed to be the same as for the standard care alone arm. Clinical parameters and utility values used in the model are presented in Table 1.

A proportion of non-responders to medical treatment underwent surgery. To derive the probability of colectomy, we used the data from the study by Feagan et al. [12] estimating that during the 52-week period, 3.68 % (15 per 408.1 patient-years) and 4.75 % (11 per 231.7 patient-years) of patients treated with adalimumab/standard care and standard care alone have a colectomy, respectively. Using the above data as our basis, we calculated the probability of a colectomy in 1 cycle (Table 1).

Patients undergoing surgery either achieved post-surgery remission and maintained it through the whole time horizon or suffered from immediate post-surgery complications, which were assumed to occur during the same cycle as surgery and resolved in 8-week period. The probability of surgery complications was calculated based on the study by Arai et al. [9] and Fazio et al. [8] (Table 1). After resolving the complications within the 8-week period, it was assumed that patients achieve post-surgical remission, just as patients who did not experience any complications.

### Costs

Costs were considered from a national health system (National Health Fund, NHF) perspective and from social perspective, and therefore, direct medical and indirect costs were included. The costs were presented in 2015 Polish zloty (PLN), and the results were presented in Euros (€; €1 = 4.2 PLN). Direct medical costs considered in the model included those related to initiation and maintenance treatment with adalimumab (drug and administration costs), standard care, monitoring and hospitalization costs, surgery costs, and treatment of complications after surgery costs.

The costs of drugs used in the study population are based on the actual unit prices of reimbursement medical products. Table 2 presents all drugs’ unit costs. The dosage of drugs used in standard care, as well as monitoring costs was determined by expert opinion.

Indirect costs come from study carried out in Poland on 202 patients with UC (unpublished). They include absenteeism, presenteeism, and costs of leaving earlier the labor market, separately for remitted patients and those with active disease. Indirect costs generated by patients in remission were assigned to responders (patients with respond or remission), and indirect costs generated by patients with active disease were assigned to the rest.

### Utilities and quality of life

A systematic review was made to identify the utility values for different health states in the model. After analysis of the available data, we chose the values presented by Woehl et al. [10] because this study is the most useful for source utility values of different stages included in the model; it reported EQ-5D utility values and was carried out on 18,573 patients from the UK (Table 1). The utility values were reported for following states: remitting disease, mild disease, and moderate to severe disease. These categories of disease severity were based on the simple colitis activity index. We assumed that the utility value for moderate to severe disease that responded to treatment was equal to the value for mildly active disease by Woehl et al. [10]. In patients during the treatment or who are in an active disease state or had complications after surgery, the utility value was assumed to be as in active moderate to severe disease. We assumed that in the post-surgery remission state, the utility value would be lower than that in the remission after the treatment state, which reflects the effect of chronic complications after a colectomy on the patient’s quality of life. All utility values are presented in Table 1.

An alternative set of utility values was used in the sensitivity analyses, based on the study by Arseneau et al. [11]. There was no change in the sensitivity analyses in utility value for remission after surgery state; to all other states, different utility values were assigned and are presented in Table 1.

### Economic analysis

The primary outcome of this simulation study was the ICUR of the treatment with adalimumab/standard care and the standard care alone, expressed as an incremental cost per QALY saved. The ICUR was calculated by dividing the difference in total costs (from the public payer’s and social perspective) by the difference in effectiveness in QALYs between adalimumab/standard care and standard care alone.

### Sensitivity analysis

Sensitivity analyses to evaluate the robustness of our findings were conducted. Variability of cost-effectiveness results according to the change of key variables was assessed using one-way sensitivity analysis. Values used in sensitivity analysis for clinical, cost, and utility parameters are presented in Table 1 and 2. Parameter uncertainty was evaluated using probabilistic sensitivity analysis (PSA).

## Results

### Base-case analysis

The results of the base-case analysis are presented in Table 3. The treatment of UC patients with adalimumab/standard care instead of the standard care alone resulted in 0.140 additional years of life in full health.

**Table 3:** Base-case results

End point	Adalimumab + standard care	Standard care alone	Incremental value
QALY	15.204	15.064	0.140
Total direct costs - public payer perspective^a^	€20,598	€9950	€10,647
Adalimumab costs^b^	€10,550	€0	€10,550
Standard care costs	€5328	€5247	€82
Monitoring costs	€3196	€3149	€47
Colectomy costs	€1523	€1555	€-32
Total indirect costs	€73,168	€73,820	€-652
Total direct and indirect costs - social perspective^c^	€93,765	€83,770	€9995
ICUR - public payer perspective	76,120 €/QALYG		
ICUR - social perspective	71,457 €/QALYG		

€1 = 4.2 PLN, based on the average exchange course from the year 2015

*€* euro

^a^Total direct costs include: pharmacotherapy costs (biological treatment), standard care costs, monitoring costs, adalimumab administration costs, colectomy and complications after surgery costs

^b^Drug and administration costs

^c^Total indirect costs included absenteeism, presenteeism, cost of early leaving the labor market

The treatment with adalimumab/standard care was found to be more expensive than treatment with the standard care alone from the NHF perspective by €10,647 and from the social perspective by €9995. The incremental cost per QALY gained was €76,120 from NHF perspective and €71,457 from social perspective (Table 3.).

### Sensitivity analysis

Results of various one-way sensitivity analyses are presented in Supplementary materials. The range of ICUR values obtained during the one-way sensitivity analysis was from 38,924 €/QALYG to 265,081 €/QALYG from NHF perspective and from 34,244 €/QALYG to 260,504 €/QALYG from social perspective. Biological treatment with no time restriction (until disease progression, i.e. lose of response, or death) resulted in ICUR value equals 97,672 €/QALYG from NHF perspective and 92,448 €/QALYG from social perspective. The difference in QALY between adalimumab/standard care and standard care alone was 0,480 with assumption of no time limitation of biological treatment.

**Fig. 2:**
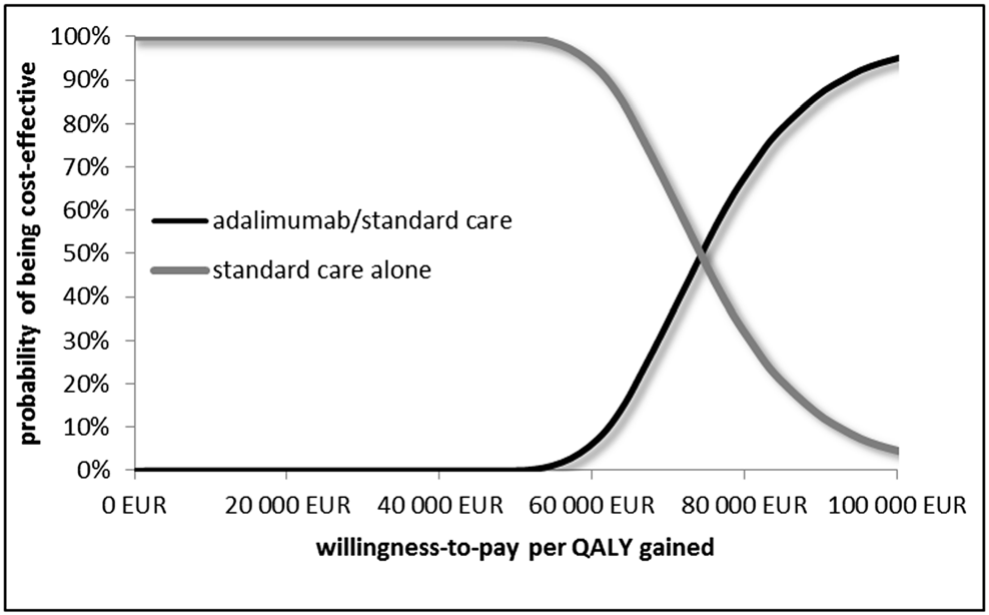
Cost-effectiveness acceptability curve showing the probability that adalimumab with standard care is cost-effective vs. standard care alone at a range of different threshold values

The results of the PSA, testing the whole range of all the uncertain parameters, are presented as a cost-effectiveness acceptability curve (Fig. 2). The mean ICUR and the 95 % confidence interval for adalimumab/standard care when compared to the standard care alone was 73,909 €/QALYG, 95 % CI: 56,745–107,058 from the public payer’s perspective and 69,270 €/QALYG, 95 % CI: 52,132–102,190 from social perspective. The results of the PSA suggest adalimumab/standard care to be cost-effective with a WTP equals about €73,800.

## Discussion

Using a 30-year time horizon and the restriction for the duration of TNFα inhibitor therapy to 1 year, adalimumab/standard care treatment turned out to be more effective and more costly option compared with the standard care alone in Poland. One year biologic treatment provided an ICUR value of 71,457–76,120 €/QALYG, depending on the perspective. Biologic treatment came to be more effective but less cost-effective for the public payer and society when there is no restriction for treatment duration.

The present economic model is the first study which assesses the biological maintenance treatment of UC in Poland and is the first study which included indirect costs of the disease. We used the data from randomized clinical trial to assess the effectiveness of biological treatment, as there is no data concerned Polish patients. Such a model can offer support to the decision makers as long as it reflects real-world conditions.

Even though the present study evaluated the cost-effectiveness of adalimumab in the Polish setting, the results could be adopted by the healthcare system in other countries. To use this economic evaluation in other country, it should be checked: (1) if the patients’ characteristics are similar, (2) if the comparator corresponds to the clinical practice in this country, and (3) if the treatment pattern is similar [13]. Additionally, the country-specific costs of drug and medical procedures should be included.

In our analysis, we did not include mortality due to UC because there is an evidence which indicates that patients with UC have normal life expectancy, subsequently, meaning that treatment will not influence the survival. Additionally, mortality was not an outcome in the reference clinical study. We also did not include treatment-related adverse events, as they have a relatively small impact on the cost and quality of life. Some assumptions had to be made concerning the utility values. The utility value for mildly active disease was assigned to patients who responded to treatment, while in patients who had complications after surgery, the utility value was assumed to be as that in active moderate to severe disease.

A systematic review by Xie carried out in October 2014 was found, concerned the economics of adalimumab for ulcerative colitis [14]. Author identified three economic analysis for adalimumab in UC. Only one of them (Ali et al. [15]) compared adalimumab/standard care with standard care alone. This study is available only in a form of abstract, and limited information about the methodology is provided. The results were presented only in a form of ICUR values, which were for adalimumab vs. standard care £96,733 and £22,087 over 1-year and 5-year horizons (2010 values), respectively. It was impossible to compare the results of above study with ours as limited information about the model inputs, assumptions, and structure is provided in the abstract. We also performed our own review of published economic analysis for study subject. We found two additional studies for adalimumab/standard care compared with standard care alone in UC. Both studies were presented in a form of abstract; full texts are not available [16, 17]. No results concerning the effectiveness were presented. The ICUR values obtained in the above studies for adalimumab/standard care vs. standard care alone were C$96,812 over 5-year horizon (2013 values) [16] and €46,815 over 10-year horizon (2013 values) [17]. As in the case of previous study, it is hard to compare our results with the above because only abstracts with limited information about methodology, inputs, and assumptions are available. All three published economic analyses showed that adalimumab/standard care compared with standard care alone in UC seems to be a cost-effective treatment option. It is worth to mention that none of the identified studies included indirect costs.

## Electronic supplementary material

ESM 1 (DOCX 17 kb)
