# Supplementary material for: Cost-utility analysis of 1-year treatment with adalimumab/standard care and standard care alone for ulcerative colitis in Poland
Source: Eur J Clin Pharmacol. 2016 Aug 6;72(11):1319–25. doi: 10.1007/s00228-016-2103-4 (PMC5055904; doi:10.1007/s00228-016-2103-4)
Supplement: Supplementary file 1 — (DOCX 17 kb) [file 228_2016_2103_MOESM1_ESM.docx]

**Table. Sensitivity analysis results.**

| **Parameter** | **ICUR [**€**/QALYG]** | |
| --- | --- | --- |
|  | **Public payer perspective** | **Social perspective** |
| Time horizon - lower value (10 years) | 77,711 | 70,698 |
| Time horizon - upper value (50 years) | 76,001 | 71,485 |
| Discount rate for outcomes - lower value (0%) | 74,377 | 69,820 |
| Discount rate for outcomes - upper value (5%) | 76,847 | 72,140 |
| Discount rate for costs - lower value (0%) | 76,496 | 72,220 |
| Discount rate for costs - upper value (10%) | 75,794 | 70,856 |
| Body weight - lower value (73.60 kg) | 76,115 | 71,451 |
| Body weight - upper value (77.14 kg) | 76,126 | 71,463 |
| Response - 8. week, adalimumab/standard care (RR) - lower value | 76,478 | 72,102 |
| Response - 8. week, adalimumab/standard care (RR) - upper value | 75,713 | 70,723 |
| Response - 8. week, standard care alone - lower value | 72,826 | 68,233 |
| Response - 8. week, standard care alone - upper value | 79,675 | 74,936 |
| Remission - 8. week, adalimumab/standard care (RR) - lower value | 265,081 | 260,504 |
| Remission - 8. week, adalimumab/standard care (RR) - upper value | 38,924 | 34,244 |
| Remission - 8. week, standard care alone - lower value | 103,495 | 98,865 |
| Remission - 8. week, standard care alone - upper value | 58,725 | 54,041 |
| Response - 52. week, adalimumab/standard care (RR) - lower value | 76,120 | 71,457 |
| Response - 52. week, adalimumab/standard care (RR) - upper value | 76,120 | 71,457 |
| Response - 52. week, standard care alone - lower value | 75,780 | 71,106 |
| Response - 52. week, standard care alone - upper value | 76,576 | 71,927 |
| Remission - 52. week, adalimumab/standard care (RR) - lower value | 76,120 | 71,457 |
| Remission - 52. week, adalimumab/standard care (RR) - upper value | 76,120 | 71,457 |
| Remission - 52. week, standard care alone - lower value | 69,617 | 64,974 |
| Remission - 52. week, standard care alone - upper value | 84,864 | 80,172 |
| Complications after surgery - lower value | 76,172 | 71,503 |
| Complications after surgery - upper value | 76,120 | 71,457 |
| Utility weight, active treatment - lower value | 68,128 | 63,954 |
| Utility weight, active treatment - upper value | 76,120 | 71,457 |
| Utility weight, remission - lower value | 99,482 | 93,387 |
| Utility weight, remission - upper value | 70,595 | 66,270 |
| Utility weight, remission after surgery - lower value | 76,120 | 71,457 |
| Utility weight, remission after surgery - upper value | 76,120 | 71,457 |
| Utility weight, complications after surgery - lower value | 76,120 | 71,457 |
| Utility weight, complications after surgery - upper value | 76,148 | 71,483 |
| Utility weight, response - lower value | 76,120 | 71,457 |
| Utility weight, response - upper value | 97,672 | 92,448 |
| Maximal treatment duration - lower value (12 months) | 82,631 | 77,569 |
| Maximal treatment duration - upper value (360 months) | 70,561 | 66,238 |
| Surgery rate (RR) - lower value | 76,270 | 72,156 |
| Surgery rate (RR) - upper value | 75,753 | 69,739 |

€ - euro, €1 = 4.2 PLN, based on the average exchange course from the year 2015; RR - relative risk; QALYG - Quality adjusted life years gained; ICUR - incremental cost-utility ratio.
